# The AST/ALT ratio predicts survival and improves oncological therapy decisions in patients with non-small cell lung cancer receiving immunotherapy with or without radiotherapy

**DOI:** 10.3389/fonc.2024.1389804

**Published:** 2024-08-26

**Authors:** Yanyan Zhang, Jingxin Zhang, Shijie Shang, Jiachun Ma, Fei Wang, Meng Wu, Jinming Yu, Dawei Chen

**Affiliations:** ^1^ Department of Shandong Provincial Key Laboratory of Precision Oncology, Shandong Cancer Hospital and Institute, Shandong First Medical University and Shandong Academy of Medical Sciences, Jinan, Shandong, China; ^2^ Shandong First Medical University and Shandong Academy of Medical Sciences, Jinan, Shandong, China; ^3^ Department of Shandong Provincial Key Laboratory of Precision Oncology, Shandong University Cancer Center, Jinan, Shandong, China; ^4^ Cancer Center, Union Hospital, Tongji Medical College, Huazhong University of Science and Technology, Wuhan, Hubei, China; ^5^ Lung Cancer Center, West China Hospital, Sichuan University, Chengdu, Sichuan, China

**Keywords:** non-small cell lung cancer, aspartate aminotransferase, alanine transaminase, AST/ALT ratio, immunotherapy without radiotherapy, immunotherapy combined with radiotherapy

## Abstract

**Background and purpose:**

Immunotherapy, with or without radiotherapy (iRT or ICIs-nonRT), is the standard treatment for non–small cell lung cancer (NSCLC). Nonetheless, the response to the treatment varies among patients. Given the established role of aspartate aminotransferase/alanine transaminase (AST/ALT) ratio in predicting cancer prognosis, we sought to identify whether the pre-treatment AST/ALT ratio has the potential to serve as a prognostic factor for NSCLC patients receiving ICIs-nonRT and iRT.

**Materials and methods:**

We retrospectively analyzed NSCLC patients who received immunotherapy between April 2018 and March 2021. Patients were classified into iRT group and ICIs-nonRT group and further classified based on AST/ALT ratio cut-off values. The Kaplan-Meier (KM) method estimated the time-to-event endpoints (progression-free survival (PFS) and overall survival (OS)

**Results:**

Of the cohort, 239 underwent ICIs-nonRT and 155 received iRT. Higher AST/ALT ratios correlated with worse outcomes in the ICIs-nonRT group but indicated better outcomes in those who received iRT. Multivariate analysis validated AST/ALT ratio as an independent prognostic factor. For AST/ALT ratios between 0.67-1.7, both ICIs-nonRT and iRT yielded similar treatment outcomes; with AST/ALT ratios greater than 1.7, iRT could be a more favorable treatment option (*P*=0.038). Conversely, for ratios less than 0.67, ICIs-nonRT could be a more favorable treatment option (*P*=0.073).

**Conclusions:**

The pre-treatment AST/ALT ratio demonstrates potential as a prognostic marker for treatment outcomes in NSCLC patients receiving either ICIs-nonRT or iRT. This finding could help guide clinicians in selecting more effective treatment protocols, thereby enhancing patient prognosis.

## 1 Introduction

Lung cancer remains the leading cause of cancer-related fatalities (1), with non-small cell lung cancer (NSCLC) accounting for approximately 85% of lung cancer cases (2). Among these, advanced NSCLC presents an especially grim prognosis, exhibiting a 5-years survival rate ranging from 10% to 30% (3).

Over time, therapeutic advances such as immunotherapy and radiotherapy have shown promise in improving outcomes for NSCLC patients (4). Although immunotherapy offers a durable response and long-term survival for a fraction of patients, resistance to this treatment is unfortunately commonplace, with hyperprogression observed in some instances (5). As a potential solution, the combination of immunotherapy and radiotherapy (iRT) is being considered as a more promising way to treat NSCLC. This combined strategy aims to enhance positive immunoregulation while significantly attenuating negative immune resistance, thereby potentially providing superior survival prognosis (6, 7). However, resistance to both radiotherapy and immunotherapy remains a challenge, leading to poor response to iRT in some patients (8). To date, a definitive biomarker guiding clinicians to judiciously apply either iRT or immunotherapy without radiotherapy (ICIs-nonRT) to the appropriate patient population has not been identified.

The aspartate aminotransferase/alanine transaminase (AST/ALT) ratio of serum levels was first proposed by Fernando De Ritis in 1957 as an indicator of hepatocellular damage or death (9), has lately been identified as a significant prognostic factor for several types of cancers, including bladder cancer (10), testicular cancer (11), hepatocellular carcinomas (12), pancreatic cancer (13) and prostate cancer (14), but there is scarce data regarding the role of AST/ALT ratio as a prognostic factor in NSCLC. Additionally, increasing number of studies have shown that reprogramming of glutamine metabolism is a putative determinant of the anti-tumor immune response in the tumor microenvironment (TME) (15). AST and ALT play crucial roles in glutamine metabolism. Malignant tumors, in order to ensure sufficient energy, exhibit increased glutamine metabolism in addition to the “Warburg effect” to sustain nucleotide biosynthesis and the synthesis of non-essential amino acids in proliferating tumor cells (16–18). Tumor cells transport glutamine into cells through specific transporters, and then convert it into glutamate under the action of glutaminase (AST, ALT and Phosphoserine Aminotransferase 1), and further convert it into α-ketoglutarate (α-KG), which enters the Tricarboxylic Acid cycle (TCA) and participates in the onset, development and dissemination of tumors (19, 20). Similar to malignant cells, immune cell activation also requires the uptake of glutamine (21). Immune cells uptake glutamine at similar or higher rates than glucose (22), with glutamine partially oxidized to CO2 within immune cells and converted to glutamate, alanine, and aspartate. This unique transformation is vital for immune cell function (15, 23). The appropriate concentration of glutamine promotes the expression of lymphocyte surface markers such as CD71, CD25, and CD45RO, as well as the production of cytokines such as IL-6, gamma-interferon (IFN-γ), and TNF-α (24–27). Glutamine metabolism also plays a major role in the activation of lymphocytes and is necessary for the differentiation of B lymphocytes into plasma cells and lymphoblasts. At the same time, glutamine is also necessary for T and B lymphocytes, for their proliferation, protein and antibody synthesis, and IL-2 production (28). Glutamine metabolism also plays a key role in regulating macrophage activation, and the synthesis and secretion of pro-inflammatory cytokines, such as IL-1, TNF-α and IL-6. In addition, α-KG produced by glutamine metabolism promotes the differentiation of M2 macrophages (29, 30).

Therefore, given the impact of glutamine metabolism on tumor immune response and the significant role of AST and ALT in this process, our aim is to explore the relationship between the easily accessible hematological marker AST/ALT ratio and the prognosis of non-small cell lung cancer patients receiving ICIs-nonRT and iRT, to aid in more precise clinical treatment.

## 2 Materials and methods

### 2.1 Study population

**Figure 1:**
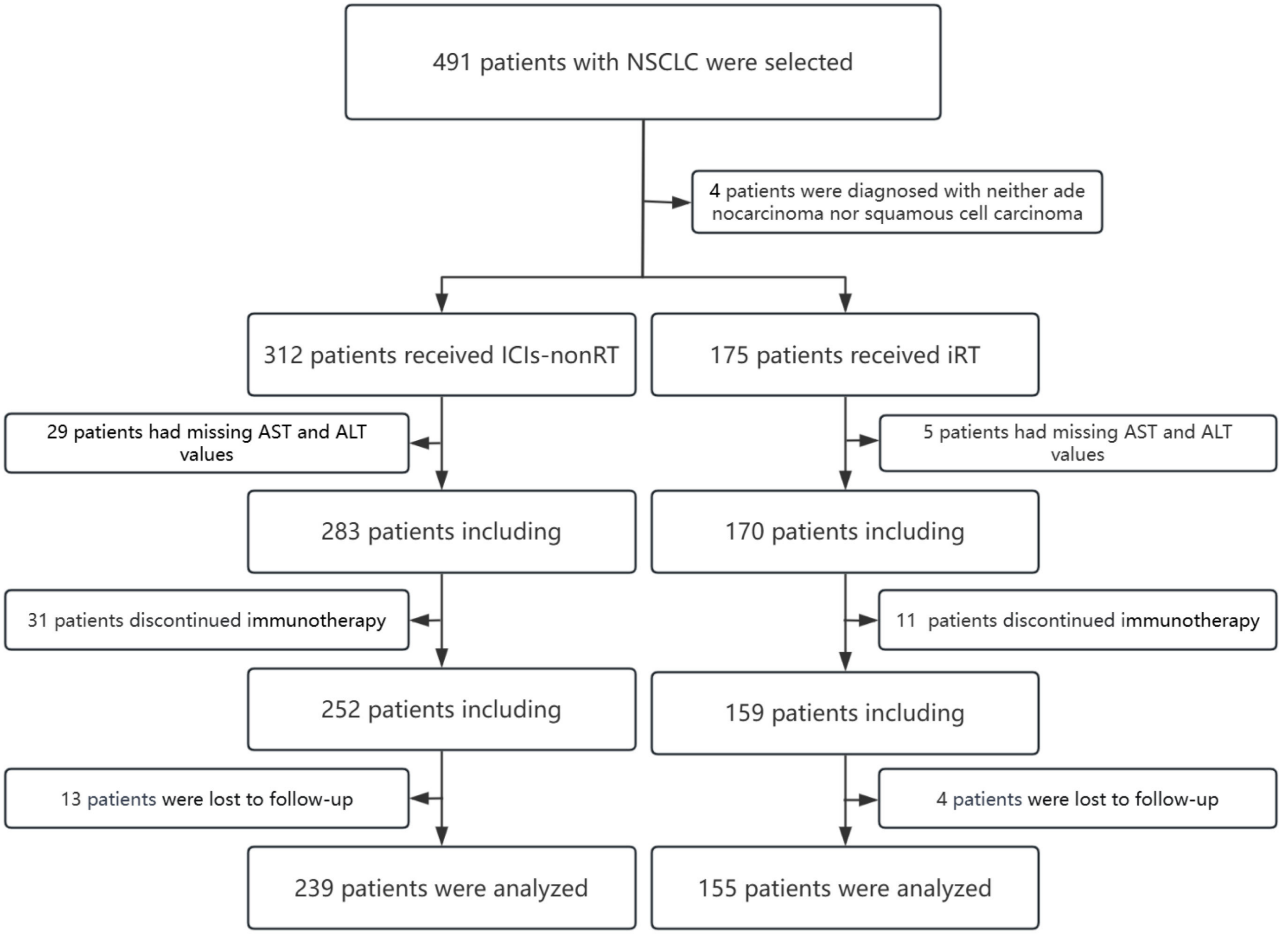
Flow chart of patient selection for this analysis. NSCLC, non-small cell lung cancer; ICIs-nonRT, immunotherapy without radiotherapy; iRT, immunotherapy combined with radiotherapy.

This retrospective study was conducted at a single institution. According to the AJCC 8th TNM and systematic staging imaging, including computed tomography (CT), positron emission tomography (PET), PET/CT, and contrast-enhanced magnetic resonance imaging, we identified 491 stage III and IV NSCLC patients who received non-operative immunotherapy between April 2018 and March 2021. Patients were then stratified into two groups: an iRT group and an ICIs-nonRT group, based on the inclusion and exclusion of radiotherapy in combination with immunotherapy. Additionally, within each group, patients were further subdivided based on the optimal cut-off values of AST/ALT ratio. Hematological indicators of AST and ALT were recorded within 5 days before the initiation of the first immunotherapy. The study excluded patients who lacked complete hematological parameters prior to their first dose of ICIs-nonRT or iRT, and those who received immunotherapy at other institutions. The detailed exclusion criteria are shown in **Figure 1**.

### 2.2 Treatment

In the ICIs-nonRT group, an overwhelming 98% of patients were treated with anti‐programmed cell death-1 (PD-1) immunotherapy, while a smaller fraction of only 2% received immunotherapy with an anti-programmed cell death ligand-1 (PD-L1) agent. Similarly, in the iRT group, a majority of 92% of patients were treated with anti‐PD-1 immunotherapy, with a marginally higher proportion of 8% received immunotherapy with an anti-PD-L1 agent. Regarding radiation therapy, within the iRT group, a predominant 96% of patients received intensity-modulated radiation therapy (IMRT), with the remainder divided between 1% receiving volumetric modulated arc therapy (VMAT) and 3% receiving three-dimensional conformal radiation therapy (3D-CRT). Detailed information on immunosuppressive agents and radiotherapy mode in each group was provided in **Supplementary Notes 1**, **2**.

### 2.3 Statistical analysis

The primary endpoint of the study was overall survival (OS), while the secondary endpoint was progression-free survival (PFS). Both OS and PFS were defined from the first day of iRT or ICIs-nonRT until the event occurrence or the last follow-up. Continuous data are presented as median with 25th-75th percentiles or mean ± standard deviation and compared with the nonparametric Mann-Whitney U test or independent samples t test. The normality of distribution of continuous data was evaluated using the Kolmogorov-Smirnov test. Categorical variables are presented as count and proportions (%) and compared using the chi-squared test. The optimal cut-off values of continuous variables were calculated using X-tile software (http://www.tissuearray.org/rimmlab) The Kaplan-Meier (KM) method estimated the time-to-event endpoints (OS and PFS) and the log-rank test compared among subgroups. Univariate Cox proportional hazard model was used to evaluate each potential predictor, and *P*-values ≤0.10 were enrolled in multivariate analysis. A trichotomy KM curve was plotted to determine the precise range of AST/ALT levels, and the reliability of the range of the identified optimal biomarkers was verified by KM curves. All the *P*-values were two sided, and *P ≤* 0.05 was considered to have a significant statistical difference.

GraphPad Prism 9 software was used to generate the KM curve. Univariate and multivariate Cox regressions were analyzed using the Xiantao academic analysis tool (www.xiantao.love). Descriptive statistics were performed using SPSS software (version 27.0; IBM Corp, Armonk, NY).

## 3 Results

### 3.1 Patient characteristics

A total of 394 eligible patients were ultimately enrolled in this study, including 239 in the ICIs-nonRT group and 155 in the iRT group. The baseline characteristics of each group are listed in **Tables 1**, **2**. The median follow-up time for the ICIs-nonRT group and the iRT group was 29 months and 30 months, respectively (*P*=0.279). The number of PFS events was 164 in the ICIs-nonRT group and 114 in the iRT group. The number of OS events was 101 in the ICIs-nonRT group and 75 in the iRT group.

**Table 1:** Baseline characteristics of all patients in the ICIs-nonRT and the iRT groups.

Characteristic	^1^ ICIs-nonRT (n=239)	^2^ iRT (n=155)	*P* value
Sex			
Male	182 (76.2%)	127 (81.9%)	0.173
Female	57 (23.8%)	28 (18.1%)	
**Age**	61 (54–66)	63 (56–67)	0.362
Pathological category			
Adenocarcinoma	153 (64.0%)	88 (56.8%)	0.150
Squamous carcinoma	86 (36.0%)	67 (43.2%)	
**^3^ BMI (kg/m2)**	23 (21–26)	24 (22–26)	0.170
Smoking behaviour			
Occasional/never	95 (39.7%)	69 (44.5%)	0.348
Frequent	144 (60.3%)	86 (55.5%)	
^4^ KPS			
≥90	143 (59.8%)	69 (44.5%)	0.003
<90 and ≥70	96 (40.2%)	86 (55.5%)	
Alcohol behaviour			
Occasional/never	142 (59.4%)	93 (60.0%)	0.908
Frequent	97 (40.6%)	62 (40.0%)	
Stage			
III	65 (27.2%)	49 (31.6%)	0.345
IV	174 (72.8%)	106 (68.4%)	
Line of therapy			
1	135 (56.5%)	125 (80.6%)	<0.001
>1	104 (43.5%)	30 (19.4%)	
**^5^ AST/ALT ratio**	1.15 (0.85-1.56)	1.14 (0.84-1.43)	0.301
Chemotherapy			
No	57 (23.8%)	51 (32.9%)	0.049
Yes	182 (76.2%)	104 (67.1%)	
Anti-angiogenic therapy			
No	178 (74.5%)	125 (80.6%)	0.156
Yes	61 (25.5%)	30 (19.4%)	
Bone metastasis			
No	178 (74.5%)	105 (67.7%)	0.147
Yes	61 (25.5%)	50 (32.3%)	
Brain metastasis			
No	203 (84.9%)	114 (73.5%)	0.005
Yes	36 (15.1%)	41 (26.5%)	
Heart disease			
No	113 (47.3%)	96 (61.9%)	0.004
Yes	126 (52.7%)	59 (38.1%)	
Liver disease			
No	143 (59.8%)	90 (58.1%)	0.309
Liver metastases	25 (10.0%)	7 (4.5%)	
Hepatic cysts	42 (17.6%)	39 (25.2%)	
Liver rupture	1 (0.4%)	0 (0%)	
fatty liver	6 (2.5%)	5 (3.2%)	
Hepatic calcifications	5 (2.1%)	6 (3.9%)	
Chronic hepatitis	3 (1.3%)	0 (0%)	
Hepatic hemangioma	4 (1.7%)	3 (1.9%)	
Hepatic cysts + liver metastases	2 (0.8%)	0 (0%)	
Hepatic cysts + hepatic calcifications	6 (2.5%)	2 (1.3%)	
Hepatic cysts + fatty liver	1 (0.4%)	2 (1.3%)	
Fatty liver + hepatic calcifications	1 (0.4%)	0 (0%)	
Hepatic cysts + hepatic hemangioma	1 (0.4%)	1 (0.6%)	

^1^ICIs-nonRT, immunotherapy without radiotherapy; ^2^iRT, immunotherapy combined with radiotherapy; ^3^BMl, Body Mass Index; ^4^KPS, Karnofsky performance status; ^5^AST/ALT, aspartate aminotransferase/alanine transaminase.

**Table 2:** Characteristics of all patients according to the level of pre-treatment AST/ALT ratio in ICIs-nonRT and iRT groups.

Characteristic	^1^ ICIs-nonRT			Characteristic	^2^ iRT		
	Low ^4^ AST/ALT ratio (n=193)	High AST/ALT ratio (n=46)	*P* value		Low AST/ALT ratio (n=21)	High AST/ALT ratio (n=134)	*P* value
**Age**	61 (54–65)	63 (58–70)	0.023	**Age**	58 (54–61)	63 (57–67)	0.002
**^3^ BMI(kg/m2)**	23.4 (21.2-26.0)	22.0 (20.5-24.5)	0.042	**BMI(kg/m2)**	25 ± 3	24 ± 3	0.029
**^5^ ALT**	18.2 (13.2-25.5)	9.7 (7.8-11.3)	<0.001	**ALT**	35.6(26.1-50.1)	14.4 (11.3-19.7)	<0.001
**^6^ AST**	18.5 (14.8-23.6)	19.2 (15.8-23.3)	0.336	**AST**	21.1 (14.9-28.7)	17.4 (14.6-21.2)	0.082
Sex				Sex			
Male	147 (76.2%)	35 (76.1%)	0.991	Male	18 (85.7%)	109 (81.3%)	0.628
Female	46 (23.8%)	11 (23.9%)		Female	3 (14.3%)	25 (18.7%)	
Pathological category				Pathological category			
Adenocarcinoma	125 (64.8%)	28(60.9%)	0.621	Adenocarcinoma	11 (52.4%)	77 (57.5%)	0.662
Squamous carcinoma	68 (35.2%)	18(39.1%)		Squamous carcinoma	10 (47.6%)	57 (42.5%)	
Smoking behaviour				Smoking behaviour			
Occasional/never	75 (38.9%)	20 (43.5%)	0.565	Occasional/never	9 (42.9%)	60 (44.8%)	0.869
Frequent	118 (61.1%)	26 (56.5%)		Frequent	12 (57.1%)	74 (55.2%)	
Alcohol behaviour				Alcohol behaviour			
Occasional/never	112 (58.0%)	30 (65.2%)	0.372	Occasional/never	12 (57.1%)	81 (60.4%)	0.774
Frequent	81 (42.0%)	16 (34.8%)		Frequent	9 (42.9%)	53 (39.6%)	
Stage				Stage			
III	56 (29.0%)	9 (19.6%)	0.196	III	4 (19.0%)	45 (33.6%)	0.183
IV	137 (71.0%)	37 (80.4%)		IV	17 (81.0%)	89 (66.4%)	
Bone metastasis				Bone metastasis			
No	150 (77.7%)	28 (60.9%)	0.018	No	14 (66.7%)	91 (67.9%)	0.910
Yes	43 (22.3%)	18 (39.1%)		Yes	7 (33.3%)	43 (32.1%)	
Brain metastasis				Brain metastasis			
No	164 (85.0%)	39 (84.8%)	0.974	No	11 (52.4%)	103 (76.9%)	0.018
Yes	29 (15.0%)	7 (15.2%)		Yes	10 (47.6%)	31 (23.1%)	
Heart disease				Heart disease			
No	90 (46.6%)	23 (50.0%)	0.681	No	14 (66.7%)	82 (61.2%)	0.631
Yes	103 (53.4%)	23 (50.0%)		Yes	7 (33.3%)	52 (38.8%)	
Liver disease				Liver disease			
No	117 (60.6%)	26 (56.5%)	0.610	No	10 (47.6%)	80 (59.7%)	0.297
Yes	76 (39.4%)	20 (43.5%)		Yes	11 (52.4%)	54 (40.3%)	

^1^ICIs-nonRT, immunotherapy without radiotherapy; ^2^iRT, immunotherapy combined with radiotherapy; ^3^BMl, Body Mass Index;

^4^ALT, alanine transaminase; ^5^AST, aspartate aminotransferas; ^6^AST/ALT, aspartate aminotransferase/alanine transaminase.

In the ICIs-nonRT group, patients were more likely to be treated with immunotherapy combined with chemotherapy, the majority of patients had a KPS≥90 (59.8%) and a higher prevalence of heart disease (including coronary artery disease, arrhythmia, pericardial disease, heart failure, valvular heart disease, and so on) was noted before the initiation of ICIs-nonRT treatment. In the iRT group, more patients received iRT as a first line of treatment, had a KPS<90 and ≥70(55.5%), and had brain metastases before iRT. By contrast, no significant difference was observed in the sex, age, pathological category, body mass index (BMI), smoking behavior, alcohol behavior, stage, AST/ALT ratio levels, anti-angiogenic therapy, bone metastasis and liver disease (including liver metastasis, hepatic cysts, fatty liver, chronic hepatitis, liver rupture, and so on) between ICIs-nonRT and iRT groups (**Table 1**).

In addition, our analysis identified that patients in both the ICIs-nonRT and iRT groups with high pre-treatment AST/ALT ratios (ICIs-nonRT group: AST/ALT ratio >1.7, n = 46; iRT group: AST/ALT ratio >0.67, n = 134) exhibited certain common characteristics: these patients were generally older and had lower BMI and ALT levels. Furthermore, in the ICIs-nonRT and iRT groups, it was observed that there were no significant differences between these patients with pre-treatment low and high AST/ALT ratios in the heart disease, liver disease, smoking habits, alcohol consumption, and AST levels, sex, pathological category, and tumor stage (**Table 2**).

### 3.2 Survival analysis according to AST/ALT ratio

**Figure 2:**
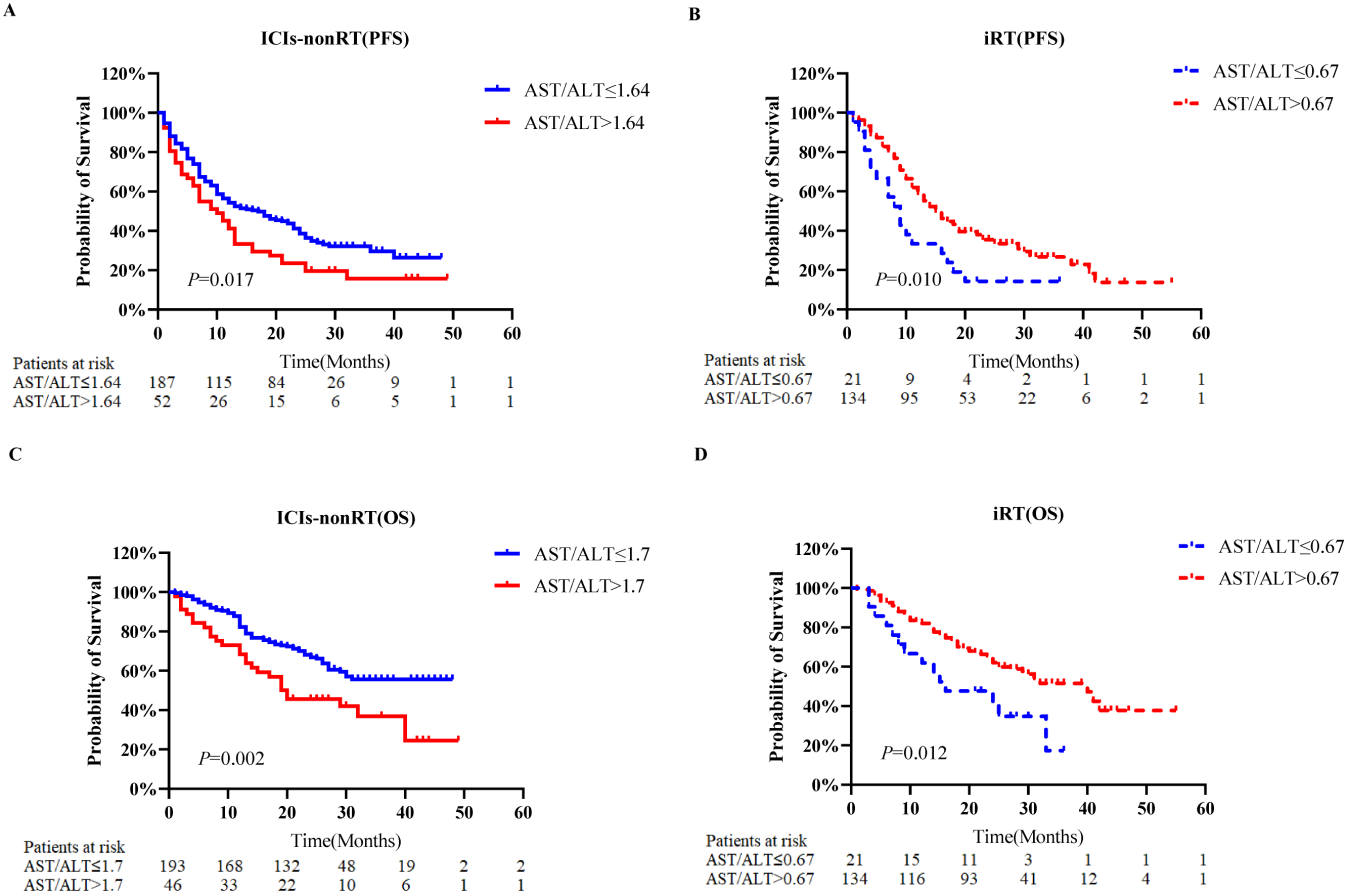
Kaplan-Meier probability plots of overall survival and progression-free survival according to the pre-treatment AST/ALT ratio category. **(A)** Progression-free survival of patients with non-small cell lung cancer who received ICIs-nonRT. **(B)** Progression-free survival of patients with non-small cell lung cancer who received iRT. **(C)** Overall survival of patients with non-small cell lung cancer who received ICIs-nonRT. **(D)** Overall survival of patients with non-small cell lung cancer who received iRT. PFS, progression-free survival; OS, overall survival; AST/ALT, aminotransferase/alanine transaminase; ICIs-nonRT, immunotherapy without radiotherapy; iRT, immunotherapy combined with radiotherapy.

**Figure 2** depicts KM analyses for PFS and OS according to the AST/ALT ratio. Patients were stratified based on the optimal AST/ALT ratio cut-off values. For the ICIs-nonRT group, patients with higher AST/ALT ratios (PFS: >1.64; OS: >1.7) were associated with worse PFS (*P*=0.017) and OS (*P*=0.002) compared to those with a lower ratio (PFS: <1.64; OS: <1.7) (**Figures 2A, C**). In contrast, in the iRT group, patients with a higher AST/ALT ratio (PFS: >0.67; OS: >0.67) had a significantly improved PFS (*P*=0.010) and OS (*P*=0.012) compared to those with lower AST/ALT ratios (PFS: <0.67; OS: <0.67) (**Figures 2B, D**).

### 3.3 Univariate and multivariate analysis of factors influencing survival

Independent predictors of PFS and OS for NSCLC patients were identified using the Cox proportional hazards regression models (**Tables 3**, **4**).

**Table 3:** Univariate and multivariate analyses of OS and PFS for different characteristics of NSCLC patients in the ICIs-nonRT group.

Variable	^1^ ICIS-nonRT								
	Univariate analysis (PFS)		Multivariate analysis (PFS)		Variable	Univariate analysis (OS)		Multivariate analysis (OS)	
	HR (95% CI)	*P* value	HR (95% CI)	*P* value		HR (95% CI)	*P* value	HR (95% CI)	*P* value
Sex					Sex				
Male	Reference				Male	Reference			
Female	0.769 (0.525-1.127)	0.179			Female	0.669 (0.402-1.114)	0.122		
Age					Age				
≤53	Reference				≤63	Reference			
>53	0.808 (0.561-1.162)	0.250			>63	1.841 (1.245-2.722)	0.002	1.962 (1.306 - 2.949)	0.001
Pathological category					Pathological category				
Adenocarcinoma	Reference				Adenocarcinoma	Reference			
Squamous carcinoma	0.930 (0.677-1.278)	0.654			Squamous carcinoma	0.809 (0.543-1.207)	0.299		
^2^ BMI(kg/m2)					BMI(kg/m2)				
≤28.34	Reference				≤23.5	Reference			
>28.34	0.721 (0.424-1.227)	0.228			>23.5	0.611 (0.408-0.917)	0.017	0.734 (0.481-1.118)	0.150
Smoking behaviour					Smoking behaviour				
Occasional/never	Reference				Occasional/never	Reference			
Frequent	0.928 (0.678-1.272)	0.644			Frequent	1.114 (0.739-1.679)	0.606		
Alcohol behaviour					Alcohol behaviour				
Occasional/never	Reference				Occasional/never	Reference			
Frequent	0.889 (0.651-1.214)	0.461			Frequent	0.893 (0.601-1.328)	0.577		
Stage					Stage				
III	Reference				III	Reference			
IV	1.471 (1.026-2.109)	0.036	1.422 (0.990-2.044)	0.057	IV	2.038 (1.224-3.393)	0.006	2.074 (1.219 - 3.530)	0.007
Line of therapy					Line of therapy				
1	Reference				1	Reference			
>1	1.472 (1.083-2.001)	0.014	1.431 (1.047-1.958)	0.025	>1	0.758 (0.506-1.134)	0.178		
^3^ AST/ALT ratio					AST/ALT ratio				
≤1.64	Reference				≤1.7	Reference			
>1.64	1.493 (1.051-2.122)	0.025	1.429 (1.001-2.040)	0.049	>1.7	1.913 (1.236-2.959)	0.004	1.738 (1.114 - 2.711)	0.015
Chemotherapy					Chemotherapy				
No	Reference				No	Reference			
Yes	0.876 (0.617-1.245)	0.461			Yes	1.278 (0.783-2.086)	0.327		
Anti-angiogenic therapy					Anti-angiogenic therapy				
No	Reference				No	Reference			
Yes	1.380 (0.983-1.939)	0.063	1.213 (0.854-1.723)	0.281	Yes	1.630 (1.069-2.485)	0.023	1.612 (1.049 - 2.478)	0.029
Bone metastasis					Bone metastasis				
No	Reference				No	Reference			
Yes	1.129 (0.801-1.592)	0.489			Yes	1.376 (0.897-2.111)	0.144		
Brain metastasis					Brain metastasis				
No	Reference				No	Reference			
Yes	1.295 (0.846-1.982)	0.235			Yes	1.663 (1.009-2.740)	0.046	1.444 (0.853-2.445)	0.171
Heart disease					Heart disease				
No	Reference				No	Reference			
Yes	1.164 (0.855-1.585)	0.334			Yes	1.328 (0.895-1.971)	0.159		
Liver disease					Liver disease				
No	Reference				No	Reference			
Yes	1.267 (0.930-1.726)	0.133			Yes	1.105 (0.744-1.642)	0.621		

^1^ ICIs-nonRT, immunotherapy without radiotherapy; ^2^ BMl, Body Mass Index; ^3^ AST/ALT, aspartate aminotransferase/alanine transaminase.

**Table 4:** Univariate and multivariate analyses of OS and PFS for different characteristics of NSCLC patients in the iRT group.

Variable	^1^ iRT								
	Univariate analysis (PFS)		Multivariate analysis (PFS)		Variable	Univariate analysis (OS)		Multivariate analysis (OS)	
	HR (95% CI)	*P* value	HR (95% CI)	*P* value		HR (95% CI)	*P* value	HR (95% CI)	*P* value
Sex					Sex				
Male	Reference				Male	Reference			
Female	0.858 (0.528-1.392)	0.535			Female	0.527 (0.262-1.058)	0.072	0.747 (0.334-1.670)	0.478
Age					Age				
≤69	Reference				≤69	Reference			
>69	1.347 (0.811-2.235)	0.250			>69	1.721 (0.961-3.081)	0.068	1.803 (0.989-3.290)	0.055
Pathological category					Pathological category				
Adenocarcinoma	Reference				Adenocarcinoma	Reference			
Squamous carcinoma	0.894 (0.615-1.300)	0.558			Squamous carcinoma	0.978 (0.619-1.546)	0.923		
^2^ BMI(kg/m2)					BMI(kg/m2)				
≤20.24	Reference				≤26.73	Reference			
>20.24	0.617 (0.344-1.105)	0.104			>26.73	1.878 (1.040-3.392)	0.037	1.628 (0.885-2.996)	0.117
Smoking behaviour					Smoking behaviour				
Occasional/never	Reference				Occasional/never	Reference			
Frequent	1.051 (0.725-1.521)	0.794			Frequent	1.548 (0.969-2.474)	0.068	1.224 (0.711-2.109)	0.466
Alcohol behaviour					Alcohol behaviour				
Occasional/never	Reference				Occasional/never	Reference			
Frequent	1.017 (0.697-1.483)	0.932			Frequent	1.393 (0.883-2.198)	0.155		
Stage					Stage				
III	Reference				III	Reference			
IV	1.360 (0.904-2.047)	0.140			IV	1.332 (0.810-2.191)	0.259		
Line of therapy					Line of therapy				
1	Reference				1	Reference			
>1	1.204 (0.765-1.896)	0.422			>1	1.449 (0.859-2.443)	0.164		
^3^ AST/ALT ratio					AST/ALT ratio				
≤0.67	Reference				≤0.67	Reference			
>0.67	0.523 (0.315-0.870)	0.012	0.516 (0.310 - 0.875)	0.011	>0.67	0.479 (0.267-0.860)	0.014	0.443 (0.240 - 0.817)	0.009
Chemotherapy					Chemotherapy				
No	Reference				No	Reference			
Yes	1.112 (0.751-1.649)	0.595			Yes	1.309 (0.790-2.168)	0.296		
Anti-angiogenic therapy					Anti-angiogenic therapy				
No	Reference				No	Reference			
Yes	1.228 (0.795-1.897)	0.355			Yes	1.177 (0.691-2.005)	0.548		
Bone metastasis					Bone metastasis				
No	Reference				No	Reference			
Yes	1.694 (1.146-2.502)	0.008	1.710 (1.157 - 2.528)	0.007	Yes	2.171 (1.353-3.484)	0.001	2.192 (1.360 - 3.533)	0.001
Brain metastasis					Brain metastasis				
No	Reference				No	Reference			
Yes	1.174 (0.783-1.761)	0.437			Yes	1.121 (0.680-1.846)	0.654		
Heart disease					Heart disease				
No	Reference				No	Reference			
Yes	1.265 (0.871-1.838)	0.217			Yes	1.279 (0.807-2.028)	0.295		
Liver disease					Liver disease				
No	Reference				No	Reference			
Yes	1.329 (0.917-1.924)	0.133			Yes	1.166 (0.739-1.838)	0.509		

^1^iRT, immunotherapy combined with radiotherapy; ^2^BMl, Body Mass Index; ^3^AST/ALT, aspartate aminotransferase/alanine transaminase.

Univariate and multivariate Cox regression analyses for the ICIs-nonRT group are presented in **Table 3**. Univariate Cox analysis identified stage, line of therapy and the AST/ALT ratio as statistically significant PFS predictors, and age, BMI, stage, the AST/ALT ratio, anti-angiogenic therapy and brain metastasis as significant OS predictors. Multivariate Cox analysis established that the pre-treatment AST/ALT ratio remained an independent prognostic factor for PFS (HR=1.429, 95% CI 1.001-2.040, *P*=0.049) and OS (HR=1.738, 95% CI 1.114-2.711, *P*=0.015). Alongside the AST/ALT ratio, line of therapy remained a significant prognostic factor for PFS, and age, stage, and anti-angiogenic therapy remained significant prognostic factors for OS according to the multivariate Cox regression analysis.

Univariate and multivariate Cox regression analyses of the iRT group are also shown in **Table 4**. Univariate Cox analysis identified the AST/ALT ratio and bone metastasis as statistically significant PFS predictor, and BMI, the AST/ALT ratio and bone metastasis as statistically significant OS predictors. Subsequent multivariate regression analysis confirmed the pre-treatment AST/ALT ratio as an independent prognostic factor for PFS (HR=0.516, 95% CI 0.310-0.875, *P*=0.011) and OS (HR=0.443, 95% CI 0.240-0.817, *P*=0.009). Furthermore, in the multivariate Cox regression analysis, in addition to the pre-treatment AST/ALT ratio, bone metastasis remained a significant prognostic factor for PFS and OS predictors.

### 3.4 Determination of the AST/ALT ratio range

**Figure 3:**
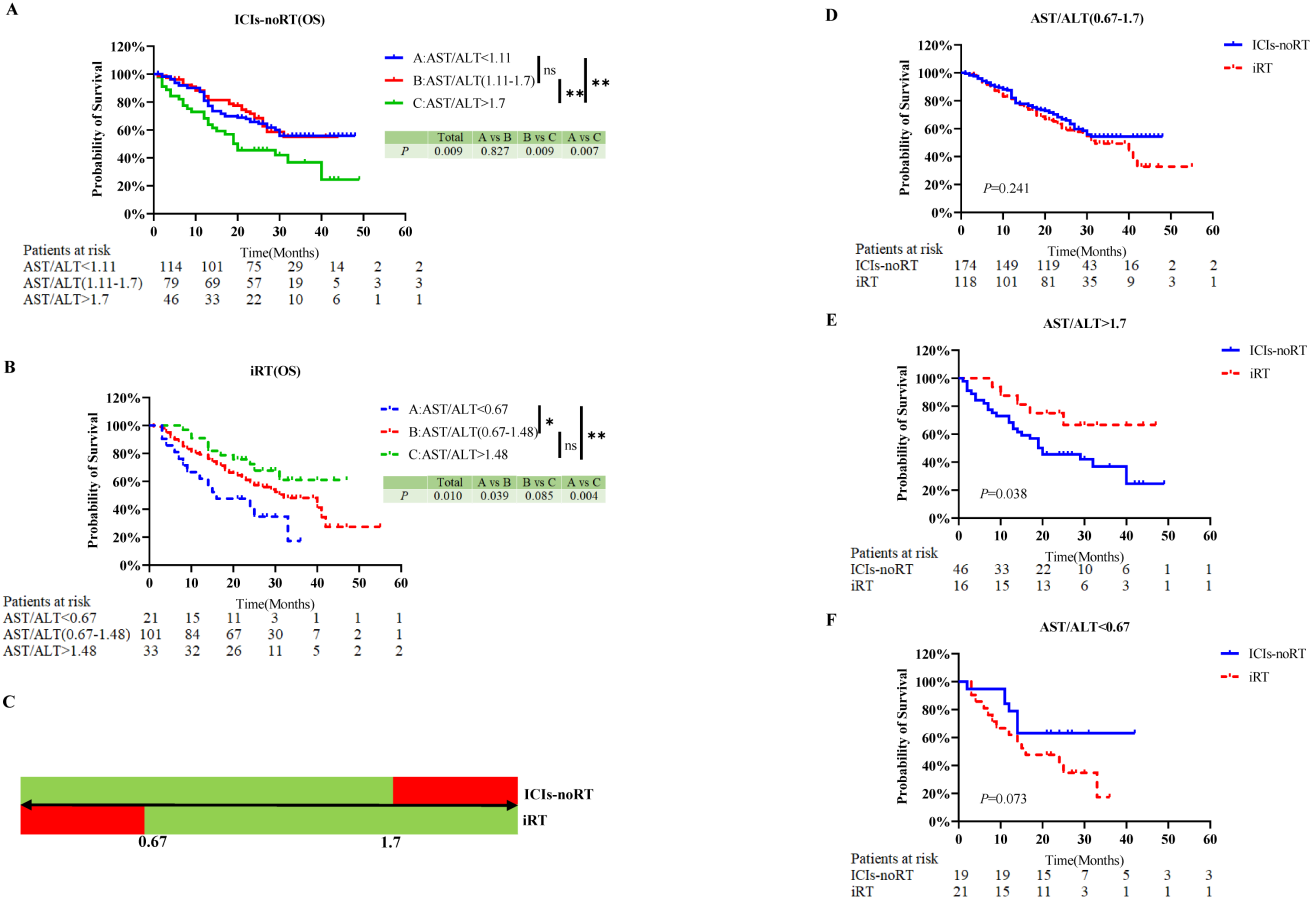
Determine the range of AST/ALT ratio in predicting OS for NSCLC patients receiving ICIs-nonRT and iRT. Kaplan-Meier probability plots of overall survival according to tertiles of the pre-treatment AST/ALT ratio: ICIs-nonRT group **(A)** and iRT group **(B)**; The schematic **(C)** summarizes the findings of Figures **(A, B)** Green indicates better patient survival. Red indicates poorer patient survival; Figures **(D–F)** validates the results of Figure **(C)** Kaplan-Meier probability plot of overall survival for patients receiving ICIs-nonRT and iRT: patients with pre-treatment AST/ALT ratios in the range of 0.67-1.7 **(D)**, pre-treatment AST/ALT ratios >1.7 **(E)** and pre-treatment AST/ALT ratios <0.67 **(F)**. OS, overall survival; AST/ALT, aminotransferase/alanine transaminase; ICIs-nonRT, immunotherapy without radiotherapy; iRT, immunotherapy combined with radiotherapy. *, *P*-value less than 0.05; **, *P*-value less than 0.01; ns, *P*-value is not significant.

To enhance oncological treatment decisions, we attempted to determine the precise range of the AST/ALT ratio (**Figures 3A, B**). The trichotomy KM curve of the AST/ALT ratio revealed that in the ICIs-nonRT group, patients with an AST/ALT ratio >1.7 had the poorest OS (*P*=0.009), while no difference was observed between the subgroup of patients with an AST/ALT ratio ranging from 1.11 to 1.7 and <1.11 (**Figure 3A**). In contrast, in the iRT group, patients with an AST/ALT ratio <0.67 had the worst OS (*P*=0.010), and no difference was found between the subgroup of patients with an AST/ALT ratio ranging from 0.67 to 1.48 and >1.48 (**Figure 3B**). To better elucidate our findings (**Figures 3A, B**), a schematic was plotted in **Figure 3C**. This schematic reveals that patients with an AST/ALT ratio ranging from 0.67 to 1.7 experienced a similar prognosis under ICIs-nonRT and iRT for NSCLC. To further confirm the reliability of the range, we validated the above results using the KM curve (**Figures 3D–F**) and reached the same conclusion. The KM curve indicated that for patients with a pre-treatment AST/ALT ratio ranging from 0.67 to 1.7, survival curves of ICIs-nonRT and iRT closely overlapped, indicating that both treatment modalities offer equivalent therapeutic effects in patients with NSCLC (**Figure 3D**). For patients with a pre-treatment AST/ALT ratio >1.7, iRT was associated with a better prognosis (*P*=0.038) than ICIs-nonRT (**Figure 3E**). For patients with a pre-treatment AST/ALT ratio <0.67, despite the comparison between ICIs-nonRT and iRT (*P*=0.073) not reaching statistical significance, a clear trend could be discerned (**Figure 3F**).

In addition, this trend could also be observed when predicting PFS based on the AST/ALT ratio (**Supplementary Figure 1**). The KM curve suggested that for patients with an AST/ALT ratio ranging from 0.67 to 1.64, both iRT and ICIs-nonRT resulted in comparable prognosis for patients with NSCLC (**Supplementary Figure 1D**). However, for patients with an AST/ALT ratio >1.64 and <0.67, no statistically significant difference was found between ICIs-nonRT and iRT (**Supplementary Figures 1E, F**). This lack of significant difference might be attributed to mechanisms of immune checkpoint inhibitors, which combat tumors by modulating tumor growth kinetics rather than solely through direct cytotoxic effects (31), leading to meaningful improvements in OS with minimal or no enhancements in PFS.

## 4 Discussion

Although numerous studies link elevated levels of AST/ALT ratio with decreased disease control and survival across different tumor types (10–14), the AST/ALT ratio’s impact in NSCLC patients receiving immunotherapy remains undefined.

Our study elucidated that high AST/ALT ratio correlated with a worse prognosis in patients receiving ICIs-nonRT, yet conversely associated with a more favorable prognosis in those receiving iRT. Additionally, we leveraged stratified Kaplan-Meier curves based on the AST/ALT ratio to demonstrate that this ratio could aid clinicians in applying either ICIs-nonRT or iRT more effectively for specific patient populations.

Prior research delineated the relationship between the AST/ALT ratio and patient prognosis, revealing that an elevated pre-treatment AST/ALT ratio was linked to a poor prognosis (32). Our data echoed these findings, indicating that NSCLC patients with a high pre-treatment AST/ALT ratio (>1.7) undergoing ICIs-nonRT treatment tended to have a poorer prognosis. However, the precise mechanisms connecting a high AST/ALT ratio and poor prognosis remain speculative, though theories regarding glutamate metabolism may offer some explanations. Glutaminolysis, a metabolic process prevalent in all proliferating cells, especially in tumor cells (18), involves conversion of glutamine to glutamate, catalyzed by Glutaminase (GLS) (33). This transformation allows cancer cells to replenish the tricarboxylic acid cycle (TCA, Krebs cycle) with α-KG as carbon source. Glutamate can be further transformed to α-KG through three aminotransferase pathways, namely ALT, AST, and phosphoserine aminotransferase1 (PSAT1). Each pathway generates a unique amino acid byproduct in addition to α-KG, with ALT being crucial in α-KG production (34, 35). Previous studies suggested that aggressive cancer cells, known for their enhanced metabolic rate, demonstrate lower serum ALT levels compared to their less invasive counterparts, likely due to increased ALT consumption (36). Besides, theories suggest that glucose metabolism and anaerobic glycolysis, wherein AST plays a significant role, might also underpin these observations. Such metabolic adaptations, known as the Warburg effect, may result in an elevated AST/ALT ratio. Nevertheless, a comprehensive understanding of these mechanisms warrants further research.

Our study also identified a unique prospective relationship between the AST/ALT ratio and prognosis. Specifically, NSCLC patients with a pre-treatment AST/ALT ratio exceeding 1.7 undergoing iRT treatment demonstrated a better prognosis compared to those with a lower AST/ALT ratio. This novel observation has rarely been reported in previous studies, and the specific mechanism is not yet clear. Previous studies have shown that radiation may stimulate resident immune cells (37, 38) and promote the influx of circulating immune cells into the tumor microenvironment (39). Similar to malignant cells, T cell activation requires glutamine uptake, and glutamine blockade inhibits oxidative and glycolytic metabolism in cancer cells, leading to decreased hypoxia, acidosis, and nutrient depletion. In contrast, the response of effector T cells to glutamine antagonists is characterized by a significant upregulation of oxidative metabolism and the adoption of a long-lived, highly activated phenotype (21). Therefore, we speculate that the better prognosis of patients with a pre-treatment AST/ALT ratio exceeding 1.7 after receiving iRT may be due to their lower ALT levels, which reduce glutamine metabolism, promote T cell proliferation and activation, reverse the inhibitory immune microenvironment, and enhance the anti-tumor immune response. Further, glutamine deprivation in cancer cells might augment oxidative stress response and reactive oxygen species (ROS) generation, leading to DNA damage and enhanced radiosensitivity (40). However, for patients with a pre-treatment AST/ALT ratio below 0.67, iRT showed a poor prognosis compared to ICIs-nonRT. While the mechanisms underlying this shift remain ambiguous, it has been speculated that radiation-resistant cells characterized by low glycolysis, reduced mitochondrial respiration, decreased TCA cycle activity, elevated glutamine anabolism might contribute to a lower AST/ALT ratio (41). However, the exact mechanisms necessitate further exploration.

Another important finding from our study involves patients with an AST/ALT ratio between 0.67 and 1.7, where ICIs-nonRT and iRT demonstrated identical prognosis in NSCLC patients. However, the detailed mechanism is still unclear and requires further investigation.

Despite our study’s novel insights into tailoring treatment modalities based on the AST/ALT ratio in NSCLC patients, it bears several limitations. These encompass potential unknown confounders and selection bias associated with retrospective studies, unidentified causes of liver enzyme alterations, uncertainty regarding the full extent of underlying disease in our study cohort, and a single pre-treatment measurement of aminotransferases. Therefore, we cannot ensure that all abnormalities truly indicate disease states. Finally, future prospective studies and external validation are necessary to determine the optimal cut-off of the AST/ALT ratio.

## 5 Conclusion

In this study, we identified the pre-treatment AST/ALT ratio as a reliable prognostic factor for survival in NSCLC patients receiving ICIs-nonRT and iRT. Interestingly, our findings challenge the conventional view that high AST/ALT ratios correlated with poor prognosis. For NSCLC patients treated with ICIs-nonRT, high AST/ALT ratios signified a poorer prognosis. Contrarily, a completely different scenario was observed in patients receiving iRT, where high AST/ALT ratios were linked to a favorable prognosis. These findings indicate that the AST/ALT ratio could serve as a valuable tool in customizing treatment for NSCLC patients. When the serum AST/ALT ratio of patients ranges from 0.67 to 1.7, ICIs-nonRT and iRT treatment seem to yield comparable outcomes. However, when the serum AST/ALT ratio is greater than 1.7, iRT appeared to be a more advantageous treatment compared to ICIs-nonRT. Conversely, ICIs-nonRT showed a superior outcome for patients with an AST/ALT ratio below 0.67 compared to iRT.

## Data Availability

The original contributions presented in the study are included in the article/**Supplementary Material**. Further inquiries can be directed to the corresponding author/s.

## Glossary

*NSCLC*: Non-small cell lung cancer
*AST/ALT*: Aspartate aminotransferase/alanine transaminase
*iRT*: Immunotherapy combined with radiotherapy
*ICIs-nonRT*: Immunotherapy without radiotherapy
*PFS*: Progression-free survival
*OS*: Overall survival
*CT*: Computed tomography
*PET*: Positron emission tomograph
*PD-1*: Programmed cell death-1
*PD-L1*: Programmed cell death ligand-1
*IMRT*: Intensity-modulated radiation therapy
*VMAT*: Volumetric modulated arc therapy
*3D-CRT*: Three-dimensional conformal radiation therapy
*BMI*: Body mass index
*KM*: Kaplan-Meier
*GLS*: Glutaminas
*TCA*: *Krebs cycle:* Tricarboxylic acid cycle
*α-KG*: α-ketoglutarate
*PSAT1*: Phosphoserine aminotransferase 1
*ROS*: Reactive oxygen species
IFN-γ: γ-interferon
TME: Tumor microenvironment
