# Supplementary material for: The AST/ALT ratio predicts survival and improves oncological therapy decisions in patients with non-small cell lung cancer receiving immunotherapy with or without radiotherapy
Source: Front Oncol. 2024 Aug 26;14:1389804. doi: 10.3389/fonc.2024.1389804 (PMC11381249; doi:10.3389/fonc.2024.1389804)
Supplement: Supplementary file 3 [file Image1.pdf]

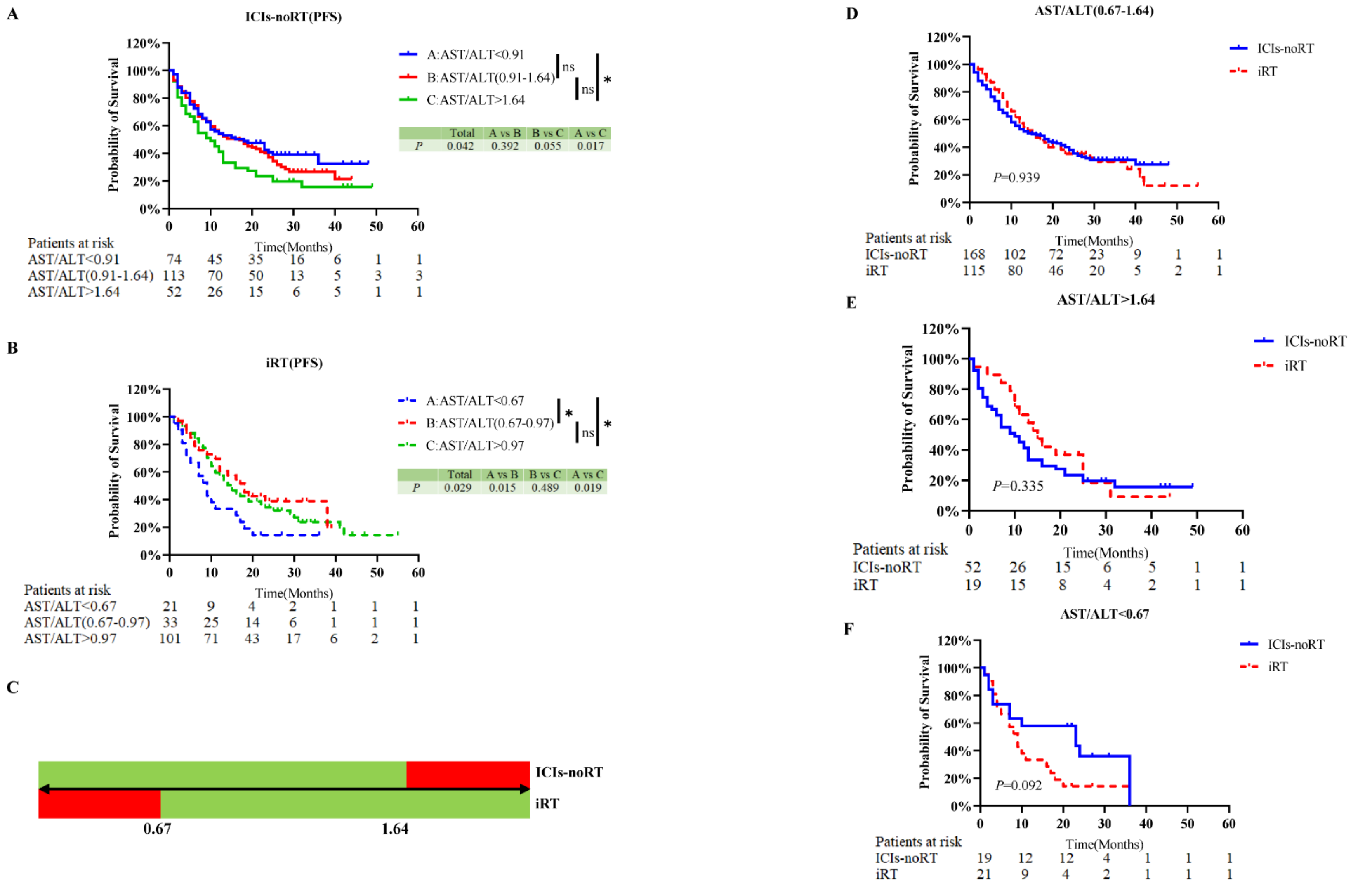

A
ICIs-noRT(PFS)
Probability of Survival
120%
100%
80%
60%
40%
20%
0%
0 10 20 30 40 50 60
Time(Months)
A:AST/ALT<0.91
B:AST/ALT(0.91-1.64)
C:AST/ALT>1.64
ns
ns
*
| | Total | A vs B | B vs C | A vs C |
| P | 0.042 | 0.392 | 0.055 | 0.017 |
Patients at risk
AST/ALT<0.91 74 45 35 16 6 1 1
AST/ALT(0.91-1.64) 113 70 50 13 5 3 3
AST/ALT>1.64 52 26 15 6 5 1 1
B
iRT(PFS)
Probability of Survival
120%
100%
80%
60%
40%
20%
0%
0 10 20 30 40 50 60
Time(Months)
A:AST/ALT<0.67
B:AST/ALT(0.67-0.97)
C:AST/ALT>0.97
*
ns
*
| | Total | A vs B | B vs C | A vs C |
| P | 0.029 | 0.015 | 0.489 | 0.019 |
Patients at risk
AST/ALT<0.67 21 9 4 2 1 1 1
AST/ALT(0.67-0.97) 33 25 14 6 1 1 1
AST/ALT>0.97 101 71 43 17 6 2 1
C
ICIs-noRT
iRT
0.67
1.64
D
AST/ALT(0.67-1.64)
Probability of Survival
120%
100%
80%
60%
40%
20%
0%
0 10 20 30 40 50 60
Time(Months)
ICIs-noRT
iRT
P=0.939
Patients at risk
ICIs-noRT 168 102 72 23 9 1 1
iRT 115 80 46 20 5 2 1
E
AST/ALT>1.64
Probability of Survival
120%
100%
80%
60%
40%
20%
0%
0 10 20 30 40 50 60
Time(Months)
ICIs-noRT
iRT
P=0.335
Patients at risk
ICIs-noRT 52 26 15 6 5 1 1
iRT 19 15 8 4 2 1 1
F
AST/ALT<0.67
Probability of Survival
120%
100%
80%
60%
40%
20%
0%
0 10 20 30 40 50 60
Time(Months)
ICIs-noRT
iRT
P=0.092
Patients at risk
ICIs-noRT 19 12 12 4 1 1 1
iRT 21 9 4 2 1 1 1

Supplementary Figure 1: Determine the range of AST/ALT ratio in predicting PFS for NSCLC patients receiving ICIs-nonRT and iRT

Kaplan-Meier probability plots of progression-free survival according to tertiles of the pre-treatment AST/ALT ratio: ICIs-nonRT group (**A**) and iRT group (**B**); The schematic (**C**) summarizes the findings of Figures A and B. Green indicates better patient survival. Red indicates poorer patient survival; Figures **D-F** validates the results of Figure **C**. Kaplan-Meier probability plot of progression-free survival for patients receiving ICIs-nonRT and iRT: patients with pre-treatment AST/ALT ratios in the range of 0.67-1.64 (**D**), pre-treatment AST/ALT ratios >1.64 (**E**) and pre-treatment AST/ALT ratios <0.67 (**F**).

PFS = progression-free survival; AST/ALT = aminotransferase/alanine transaminase; ICIs-nonRT = immunotherapy without radiotherapy; iRT = immunotherapy combined with radiotherapy.
